# Susceptibility Genes to Plant Viruses

**DOI:** 10.3390/v10090484

**Published:** 2018-09-10

**Authors:** Hernan Garcia-Ruiz

**Affiliations:** Nebraska Center for Virology, Department of Plant Pathology, University of Nebraska-Lincoln, Lincoln, NE 68503, USA; hgarciaruiz2@unl.edu; Tel.: +1-(402)-472-3008

**Keywords:** virus susceptibility genes, antiviral defense, virus movement, gene silencing, virus resistance, virus accumulation, host factors

## Abstract

Plant viruses use cellular factors and resources to replicate and move. Plants respond to viral infection by several mechanisms, including innate immunity, autophagy, and gene silencing, that viruses must evade or suppress. Thus, the establishment of infection is genetically determined by the availability of host factors necessary for virus replication and movement and by the balance between plant defense and viral suppression of defense responses. Host factors may have antiviral or proviral activities. Proviral factors condition susceptibility to viruses by participating in processes essential to the virus. Here, we review current advances in the identification and characterization of host factors that condition susceptibility to plant viruses. Host factors with proviral activity have been identified for all parts of the virus infection cycle: viral RNA translation, viral replication complex formation, accumulation or activity of virus replication proteins, virus movement, and virion assembly. These factors could be targets of gene editing to engineer resistance to plant viruses.

## 1. Introduction

**Figure 1:**
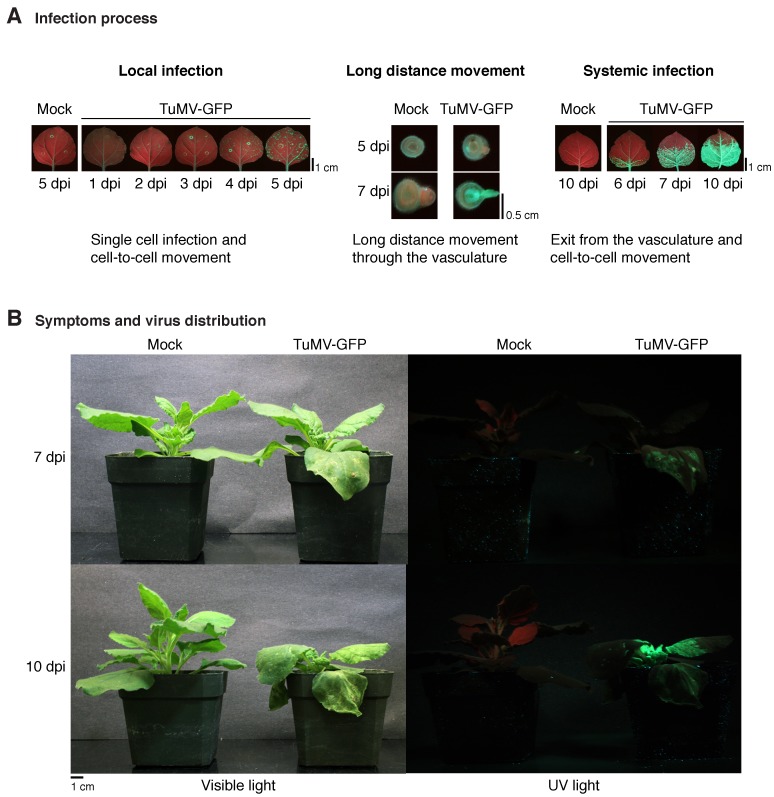
Plant virus infection progression, movement, and symptoms caused by virus infection. *Nicotiana benthamiana* plants were inoculated with GFP-tagged TuMV (TuMV-GFP) by agroinfiltration and leaves of whole plant pictures taken under visible or UV light. (**A**) Pictures showing representative local infection foci (green spots) in inoculated leaves, long-distance movement and infection of the vascular system, and progression of systemic infection in noninoculated leaves. (**B**) Symptoms of TuMV-GFP infection at 10 days post-inoculation (dpi) and distribution of virus infection as determined by UV illumination.

Viruses are molecular parasites that use cellular resources in all parts of their replication cycle. Additionally, plant viruses move cell-to-cell (local) in infected leaves and long-distance through the vascular system (systemic movement) (Figure 1A) using virus-encoded movement proteins and cellular factors. Plant viruses have been described for classes II through VII of the Baltimore classification system [1]. Accordingly, the genomes of plant viruses consist of ssDNA, dsRNA, positive-single-strand RNA, or negative-single-strand RNA. Reverse-transcribing ssRNA or dsDNA virus genomes have also been described [1]. Positive-single-strand RNA viruses are the most abundant group of plant viruses and include the genera Bromovirus, Cucumovirus, Potexvirus, Potyvirus, Tobamovirus, Tombusvirus, and others. Negative-single-strand RNA viruses include Orthotospoviruses [1].

Plant viruses are usually delivered into the cell by an insect vector and infection initiates in a single cell. Viral proteins must be translated and participate in virus replication, virion assembly, and virus movement to the neighboring cells. At every newly infected cell, the cycle is repeated [2]. After reaching the vascular system, viruses move long distances [3]. Some viruses are restricted to the vasculature. However, most viruses exit the vascular system and infect roots and young leaves away from the site of initial infection (Figure 1B). Thus, the infection process of a plant by a virus consists of a continuous cycle of virus replication at the cellular level and cell-to-cell movement [2,3].

Plant virus replication and movement are genetically determined by a combination of viral and host factors coordinated in a temporal and spatial manner [4,5,6]. Viruses express their genes through an RNA intermediate [7]. Because viruses lack ribosomes, translation of viral proteins from genomic RNA, subgenomic RNA, or mRNA is dependent on the cellular translation machinery [8,9,10].

While plant DNA viruses form minichromosomes in the nucleus of infected cells that are replicated by cellular DNA-dependent DNA polymerases [11], RNA viruses induce the formation of specialized organelle-like replication vesicles bound to cellular membranes [5,6]. These vesicles contain viral genomic RNA, viral RNA-dependent RNA polymerases, host factors and are the sites of virus replication [5,9,12,13,14]. The most detailed information about virus replication complex formation and activity is for positive-single-strand RNA brome mosaic virus (BMV), tomato bushy stunt virus (TBSV), and turnip mosaic virus (TuMV) [15,16,17]. In addition to cellular membranes, cellular proteins participate in the formation and are essential components of viral RNA replication compartments (Table 1) [5,13,14]. Other host factors modulate the accumulation or activity of virus replication proteins (Table 1).

Cell-to-cell movement of plant viruses occurs through plasmodesmata [18]. Plant viruses encode movement proteins that increase the plasmodesmata size exclusion limit or form microtubules to direct virions or nucleoprotein complexes to neighboring cells. Virus movement requires both virus-encoded proteins and cellular factors, including membranes, proteins, microtubules, or actin filaments (Table 1) [12,18,19,20,21]. Plant virus movement is reviewed in [3,22].

Plants protect themselves from viruses by several mechanisms targeting viral nucleic acids or proteins. While viral RNA and DNA are targeted by gene silencing [23], viral proteins are recognized by autophagy [24] and R-mediated innate immunity [25]. Antiviral defense restricts viral RNA translation, virus replication, movement, or virion assembly, resulting in reduced virus accumulation and/or a delay in virus movement with or without a hypersensitive response [26,27,28]. Antiviral defense mechanisms are reviewed in [26,29].

For any plant–virus combination, the outcome could be the absence of infection (incompatible interaction or nonhost) or the establishment of infection (compatible interaction or permissive host). Incompatible interactions result from the lack of cellular factors essential for the virus to replicate or move, or due to the presence of a defense mechanism restricting virus replication or movement [30,31,32]. Permissive host plants harbor necessary factors and resources and virus infection occurs through the entire plant or could be limited to inoculated leaves or the vascular system. Symptoms may or may not develop. Genetic analyses have shown that the absence of proviral factors results in the absence of infection, or reduced virus replication or movement, or both [33,34,35]. Accordingly, several terms have been used to describe these genes, such as loss-of-susceptibility, recessive resistance, or positive regulators of virus infection [33,36]. Herein, we use the term susceptibility genes, because their presence conditions virus susceptibility.

In plants, translation initiation factor eIF(iso)4E [33] and DEAD-box RNA helicase RH8 [37] illustrate susceptibility genes that are required for potyvirus infection and that are not necessary for translation of plant genes, growth, or development. This kind of genes represents opportunities to edit plant genes and engineer resistance to viruses. This review is focused on susceptibility genes to plant virus infection and the experimental systems used to identify and characterize them.

## 2. Viral Determinants of Infection

**Figure 2:**
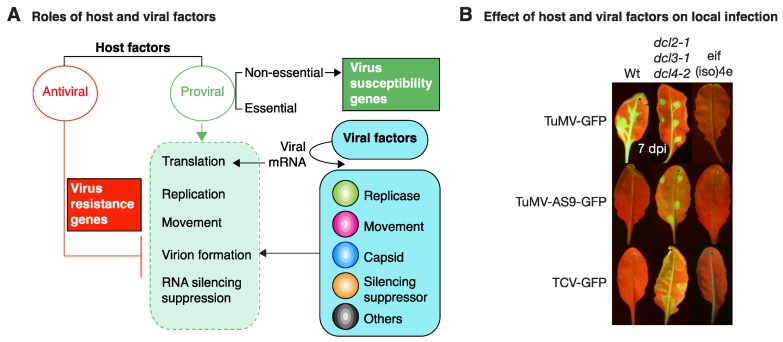
Functional groups of host and viral factors based on their role in virus infection. (**A**) Host factors may have antiviral or proviral activity. Antiviral factors (red line) condition resistance to virus infection by antagonizing one or more essential parts of the infection cycle (dotted green box). Proviral factors (green arrow) work in synchrony with viral factors in all parts of the infection cycle, determine virus susceptibility, and may be essential or nonessential to the host. (**B**) Gene silencing restricts virus infection and virus-encoded silencing suppressors are needed for infection. In the absence of translation initiation factor eIS(iso)4E, TuMV cannot infect *A. thaliana*. eIF(iso)4E is needed for potyvirus replication and/or cell-to-cell movement. *A. thaliana* leaves were mechanically inoculated with TuMV-GFP, suppressor deficient TuMV-AS9-GFP, or suppressor deficient TCV-GFP. Pictures were taken at 7 dpi under UV light.

The infection process of a plant by a virus can be divided into sequential phases: virion disassembly, viral RNA translation, viral replication complex formation, virus replication, cell-to-cell movement, systemic movement, and virion formation [3,4]. Plant viruses encode replication, movement, gene silencing suppressors, and capsid proteins (Figure 2A) that are essential for the infection process [2,38]. The absence of one or more of these virus factors results in lack of infection, reduced virus replication, or slow movement, causing low virus accumulation and mild symptoms in infected plants [39,40].

## 3. Host Genetic Determinants of Virus Infection

During the infection process, viral factors interact with host factors. Based on their role in host–virus interactions, host factors can be divided into two functional groups: antiviral and proviral (Figure 2A). Host factors with proviral activity are necessary for essential steps of the infection process, such as viral RNA translation, virus replication, movement, or virion formation (Table 1 and Figure 2A). On the contrary, host factors with antiviral activity restrict viral RNA translation, virus replication, movement, or virion formation. Viruses must evade or suppress antiviral defense responses, such as gene silencing (Figure 2B). Informative papers and reviews include [34,35,41,42,43].

At the genome-wide level, the first experimental identification of proviral and antiviral factors derived from a genome-wide screen of a yeast *(Saccharomyces cerevisiae*) deletion library for host factors affecting BMV replication [34]. Subsequently, genome-wide screens identified yeast genes with proviral or antiviral activity to TBSV [35] and to flock house virus [44]. Based on an RNA interference screen, *Drosophila melanogaster* genes were also grouped into the same functional groups with respect to the replication of influenza virus [45]. Theses genome-wide screens elegantly showed that a permissive host harbors both proviral and antiviral factors and that most of the host genes are irrelevant to virus infection.

## 4. Host Factors That Determine Virus Susceptibility

Permissive hosts contain factors required for all parts of the virus replication at the cellular level [34,35]. Additionally, permissive plants contain factors required for local and systemic virus movement [46,47]. This model predicts that in the absence of required host factors, virus accumulation is reduced at the cellular level and/or at the organismal level due to inefficient virus replication, movement, or a combination thereof. The end result is a virus-resistant phenotype characterized by reduced virus accumulation and mild symptoms with respect to susceptible plants or by the absence of infection, similar to the phenotype of a nonhost (Figure 2B). Accordingly, the presence of host factors required for virus infection or movement are genetic determinants of susceptibility to viruses.

In this review, host factors that condition susceptibility to plant viruses are organized based on sequential parts of the virus infection cycle. Susceptibility factors with essential roles in all parts of the virus infection cycle have been identified. Representative host factors are listed in Table 1 and described below. However, some host factors are involved in more than one part of the infection cycle, and for others, the exact function has not been determined.

### 4.1. Viral RNA Translation

Viruses lack ribosomes and express their genes trough mRNA [7]. Translation of all viral proteins is dependent on the cellular translation machinery, including ribosomes [8,9,10]. Accordingly, host factors that play a critical role in viral RNA translation have been identified. Using the BMV replication system in yeast, a genetic screen identified DED1, a general translation initiation factor essential for cell survival, as being necessary for BMV RNA translation [48]. Cells harboring the mutant allele DED1-18 inefficiently translate viral polymerase-like protein 2a from RNA2 [48]. Similarly, members of the deadenylation-dependent mRNA decapping complex LSM1-7 and PATH1 are required for BMV RNA1, RNA2, and RNA3 translation [10,49]. Without affecting host translation, the absence of DED1-18, LSM1-7, or PATH1 results in reduced BMV RNA replication [10,48,49].

### 4.2. Virus Replication Complex Formation

Positive-strand RNA viruses replicate in virus-induced organelle-like replication vesicles formed on intracellular membranes of the endoplasmic reticulum (ER), peroxisomes, mitochondria, or chloroplasts. This process requires the coordinated activity of viral and host factors for appropriate subcellular localization of replication proteins, membrane remodeling, lipid biosynthesis, viral RNA template selection, and formation and trafficking of replication vesicles [2,5,6]. Consistently, host factors that mediate these events have been identified (Table 1), and in mutant plants or cells lacking these essential host factors, virus replication complexes do not form or do not function normally, resulting in a drastic reduction in virus replication [54,61].

For the formation of virus replication compartments, viral proteins must localize to a cellular organelle and remodel cellular membranes to induce vesicle formation [6,12]. Several host factors have been shown to mediate these events. ER-vesicle protein 14 (ERV14) interacts with and mediates BMV 1a localization to the perinuclear ER [61], while PEX19 mediates localization of the TBSV replication proteins to the peroxisome [52]. SNF7 codes for a protein that interacts with BMV replication protein 1a and is an essential component of the replication vesicles whose membranes are permeabilized by luminal thiol oxidase ERO1 [63]. Accordingly, replication compartments do not form in mutants lacking ERV14 or SNF7 [54,61] and are dysfunctional in mutants lacking ERO1 [63].

Through interactions with 1a, membrane-shaping reticulon proteins (RHP) are critical to the formation of BMV replication compartments [55]. Similarly, through interaction with the p33 replication protein, ER-resident SNARE protein encoded by SYP81 is essential in the formation of TBSV replication compartments [59]. RAB5 encodes a GTPase protein that interacts with p33 and redistributes phosphatidylethanolamine to the replication compartments to favor TBSV replication [58].

Endosomal sorting complexes required for transport (ESCRT proteins) interact with 1a or p33 and are critical components of the BMV or TBSV replication complexes, respectively [53,54]. TuMV replication vesicles form in the ER and move to the chloroplast [113]. During TuMV infection of *Arabidopsis thaliana*, SYP71 interacts with 6K2 and mediates the fusion of virus replication vesicles to chloroplasts [56]. Bamboo mosaic virus (BaMV) replicates in chloroplast membranes. BaMV RNA associates with chloroplast phosphoglycerate kinase (chl-PGK) and is transported to the chloroplast for replication [60].

### 4.3. Accumulation or Activity of the Replication Proteins

During RNA virus replication, virus-encoded RNA-dependent RNA polymerases are responsible for positive- and negative-strand RNA synthesis. Host factors are critical components involved in template selection, accumulation, or activity of the viral RNA-dependent RNA polymerase [114]. This has been demonstrated for BMV, TBSV, and tobamoviruses (Table 1). A cytoplasmic protein related to core RNA splicing factors, LSM1, is required for efficient selection of BMV RNA templates for replication [69]. HSP70, encoding heat shock protein 70, through interactions with p27 or p33 is required for the synthesis of RNA of red clover necrotic mosaic virus (RCNMV) and TBSV, respectively [72,73,74]. Similarly, TOM1 and ARL8 are required for negative-strand synthesis of tomato mosaic virus (ToMV) [80,81], unsaturated fatty acids produced by OLE1 are needed for negative-strand synthesis of BMV RNA [70], and GAPDH regulates the asymmetrical synthesis of positive- and negative-strand RNA during TBSV replication [71].

### 4.4. Virus Movement

Plant viruses move cell-to-cell through plasmodesmata [18]. At the initial infection site, cell-to-cell movement results in the formation of local infection spots which are potentially visible (Figure 1A). After reaching the vascular system, viruses move long-distance and infect roots and young leaves (Figure 1), although some viruses remain confined to the vascular system. Cell-to-cell and long-distance movement of plant viruses is mediated by viral proteins and host factors (Table 1) [4,115,116].

Virus-encoded movement proteins modify the plasmodesmata central cavity or form tubules inside the plasmodesmata [18]. Viruses that encode tubule-forming proteins include the families Bromoviridae, Caulimoviridae, Secoviridae, and Tospoviridae. Interestingly, tubule formation is dependent on host proteins. Cauliflower mosaic virus (CaMV) moves cell-to-cell through the endocytic pathway. The CaMV MP forms tubules that pass through modified plasmodesmata and transport virions through the lumen. In the process, CaMV MP interacts with cellular prenylated Rab acceptor 1 and Rab GTPase receptor (AtRAB-F2b), which localize in early endosomes, and with plasmodesmata-located adaptins [102]. Plasmodesmata-located proteins (PDL1, -2, and -3) promote movement of grapevine fanleaf virus (GFLV) and CaMV by interacting with virus movement proteins. A mutational analysis that disrupted the interaction resulted in reduced tubule formation, delayed onset of systemic infection, and plants showing mild symptoms compared to wild-type plants [85].

Potyviruses move systemically using both xylem and phloem without forming tubules. The viral movement protein is P3N-PIPO [117]. For TuMV, and possibly other potyviruses, replication vesicles participate in cell-to-cell movement [19]. TuMV movement is dependent on PCaP1 and SEC24A through interactions with P3N-PIPO and 6K2, respectively. SEC24A interacts with 6K2 to facilitate intracellular trafficking of viral vesicles containing viral RNA. Consistent with this model, virus movement was inefficient in mutant plants lacking PCaP1 or SEC24A, resulting in reduced virus accumulation, lack of systemic movement, and mild symptoms [19]. Additionally, without affecting virus replication, potyvirus VPg-interacting protein (PVIP) is necessary for virus movement in plants through interactions with VPg. Inactivating mutations on TuMV VPg and siRNA-mediated downregulation of PVIP in *A. thaliana* abolished the interaction and resulted in reduced virus movement, accumulation, and mild symptoms [90].

*A. thaliana* synaptotagmin (SYTA) is necessary for the cell-to-cell movement of cabbage leaf curl virus (CaLCuV), turnip vein clearing virus (TVCV), and TuMV by interacting with their respective movement proteins (MP, 30K, P3N-PIPO) to alter plasmodesmata. Accordingly, in mutant plants lacking SYTA, virus infection progressed slowly and plants showed mild symptoms [91,92].

The tomato spotted wilt virus (TSWV) movement protein NSm localizes to the ER membrane and plasmodesmata and forms tubular structures that traverse the plasmodesmata. RDH3 encodes a GTP-binding motif protein that participates in the control of vesicle trafficking between ER and Golgi compartments. Consistently, cell-to-cell movement of TSVW requires the ER membrane transport system through RHD3 [47].

### 4.5. Gene Silencing Suppression

Gene silencing is an essential antiviral defense system in plants. To promote virus infection and movement, plant viruses encode suppressors of gene silencing that inhibit both endogenous and antiviral gene silencing [43]. In the absence of silencing suppressors, viruses cannot infect wild-type plants (Figure 2B) [30,39,118,119]. Interestingly, some virus-encoded gene silencing suppressors interact with and need host factors to function (Table 1). RAV2, an ethylene-inducible transcription factor, is required for suppression of gene silencing by potyviral HC-Pro and carmoviral p38 to prevent activity of primary siRNAs and for the plant malformations observed in transgenic plants expressing HC-Pro [99].

In *Nicotiana benthamiana*, the calmodulin-like protein (Nbrgs-CaM) is an endogenous plant regulator of gene silencing that functions by repressing expression of RDR6 [32]. RDR6 and SGS3 participate in the biogenesis of secondary siRNAs necessary to amplify endogenous and antiviral gene-silencing signals against RNA viruses [120,121] and geminiviruses [32]. Infection of *N. benthamiana* by tomato yellow leaf curl China virus (TYLCCNV, a geminivirus) and the associated βC1 DNA satellite induces expression of Nbrgs-CaM that in turn downregulates RDR6 expression, thus reducing antiviral defense mediated by gene silencing [32].

### 4.6. Virion Assembly and Disassembly

Upon entry into the cell, virions are disassembled prior to translation and replication. In the opposite process, after replication, viral genomes with or without RNA-dependent RNA polymerases are assembled by the capsid protein into virions [103,122]. BMV virion assembly requires replication-dependent transcription and translation of coat protein subunits [123]. Similarly, cotranslational disassembly occurs in tobacco mosaic virus (TMV) [124] and BMV [123]. These models predict that host factors are involved in virion assembly and disassembly. However, host factors necessary for virion disassembly and assembly are just beginning to be identified.

During potato virus A (PVA) infection, viral RNA is recruited away from translation into replication. This process is coordinated by the abundance and phosphorylation of the coat protein. Translation is blocked by nonphosphorylated coat protein binding to viral RNA. Detachment from the ribosomes promotes recruitment of the replication protein NIb to the 3′ UTR of the genomic RNA for the assembly of viral replication compartments. Coat protein is phosphorylated by cellular CK2. Phosphorylated coat protein interacts with ubiquitin ligase CP-interacting protein (CPIP) and HSP90. The end result is ubiquitin-mediated coat protein degradation and initiation of replication [103]. Formation of PVA virions is mediated by coat protein-dependent cotranslational inhibition of translation. When coat protein is abundant, CPIP is depleted and coat protein formed in *cis* interacts with coat protein accumulated in *trans*. The resulting complex releases the ribosomes and triggers virion assembly [122]. Plants in which CK2, CPIP, or HSP90 were individually downregulated accumulated PVA to lower levels than the wild type [103].

HC-Pro is involved in plum pox virus (PPV) assembly. PPV replication and virion formation are functionally linked [122]. In other potyviruses, host factors are required for HC-Pro silencing suppression activity [99,100]. These observations suggest that unidentified host factors are involved in potyvirus assembly through interactions with HC-Pro, NIb, or 6K2.

### 4.7. Host Factors That Condition Susceptibility by Undetermined Mechanisms

Several host factors condition susceptibility to plant viruses by mechanisms that have not been determined (Table 1). The absence of these host factors causes a reduction in virus accumulation that could result from inefficient translation, replication, movement, virion formation, or a combination thereof, as indicated in the following examples.

Eukaryotic translation elongation factor 1A (eEF1A) interacts with the 3′ UTR of TMV genomic RNA and with the replication protein 126K. Downregulation of eEF1A in *N. benthamiana* plants reduced TMV accumulation without affecting translation or the number of infection foci [64]. The effect could be mediated by reduced replication, movement, or a combination thereof.

RIM1 is a NAC transcription factor that regulates jasmonic acid signaling [106]. A genetic analysis of Tos17 insertion mutant rice lines showed that RIM1 is necessary for RDV accumulation, possibly through replication [105]. While lines overexpressing RIM1 accumulated higher levels of RDV, mutant lines lacking RIM1 did not show symptoms of infection and accumulated RDV to low levels [105]. The viral component that interacts with RIM1 has not been described and the specific part of the replication that requires RIM1 has not been identified.

The essential for potexvirus accumulation 1 (EXA1) gene contains a GYF domain and a conserved motif for interaction with eukaryotic translation initiation factor 4E (eIF4E), and is highly conserved in plants. EXA1 is required for infection by plantago asiatica mosaic virus (PlAMV, genus Potexvirus). In the absence of EXA1, PlAMV, alternanthera mosaic virus (AltMV), and PVX failed to establish infection [112].

The inositol-requiring protein-1 (IRE1) and its substrate bZIP60 are a major sensor of the unfolded protein response signaling network in plants. Infection by TuMV induces expression of bZIP60 through 6K2. In the absence of bZIP60, TuMV accumulated to reduced levels [110].

The Hsc70-2 protein of *N. benthamiana,* which is induced upon infection, interacts with the beet black scorch virus (BBSV) replication protein p23. Accordingly, downregulation of Hsc70-2 resulted in reduced BBSV accumulation [76], likely resulting from reduced virus replication.

## 5. Identification of Host Factors That Determine Virus Susceptibility

A combination of experimental approaches has been used to identify proviral host factors (Table 1). The model hosts *N. benthamiana*, *A. thaliana*, and yeast have been remarkably useful. Yeast has been used as a heterologous host to replicate BMV [34], carnation Italian ringspot virus [125], TBSV and other tombusviruses [35,42]. Yeast replication systems provided the foundation to screen at the genome-wide level for host factors necessary for virus replication [34,35,126]. Host genes with essential roles in the formation of viral RNA replication vesicles have been identified and characterized mainly using yeast to replicate BMV or TBSV (Table 1).

Based on the model that viral factors interact and may form complexes with cellular factors, yeast two-hybrid assays or immunoprecipitation of viral factors followed by mass spectrometry have led to the identification of cellular factors needed for translation [50], replication complex formation [59], viral RNA replication [37], movement [91,92], gene silencing suppression [100], and others [109] (Table 1).

In species with low or no natural variation in virus resistance, chemical mutagenesis was used to identify susceptibility factors [33,112]. Natural genetic variation and fine gene mapping in melon (*Cucumis melo* L.) led to identification of vacuolar protein sorting 41 (CmVPS41), which is conserved among plants, animals, and yeast and is required for post-Golgi vesicle trafficking towards the vacuole. CmVPS41 may participate in systemic movement, because cucumber mosaic virus (CMV) 3a interacts with CmVPS41 to promote viral entry into the phloem [98].

Transient expression systems in *N. benthamiana* have been implemented to identify or characterize viral factors that trigger an antiviral response or are necessary for virus infection [127].

## 6. Essential and Nonessential Host Factors

Factors that condition virus susceptibility may be essential or nonessential for host survival (Figure 2A). Because they are required for survival, essential genes cannot be removed from the host. Conditional repression of expression or temperature-sensitive expression was used to determine the role of yeast essential genes in BMV and TBSV replication [126,128]. This genetic analysis identified 19 essential yeast genes that antagonized and 19 essential yeast genes that were required for BMV replication [126]. Similarly found were 46 essential yeast genes that antagonized and 72 essential yeast genes that are required for TBSV replication [128]. Genes essential for yeast survival and necessary for BMV or TBSV replication participate in translation (DED1), protein homeostasis (ESS1, HSF1, PFY1), ribosome biosynthesis (NOP), cell cycle (DCD53), lipid synthesis (ALG14), RNA metabolism (RNA15, SW2), and others [126,128].

Table 1 lists representative nonessential host factors that condition susceptibility to plant viruses. These genes represent opportunities to edit plant genes and engineer resistance to viruses, as demonstrated for eIF(iso)4E [129]. In plants, translation initiation factor eIF(iso)4E is required for potyvirus infection and is dispensable for translation of plant genes, growth, and development [33]. Thus, using CRISPR/Cas9, an inactivating mutation was introduced in the eIF(iso)4E in *A. thaliana*. The resulting plants were immune to TuMV [129].

## 7. Concluding Remarks

Plant virus replication and movement are mediated by viral genetic determinants interacting with and functioning in synchrony with cellular factors. Consequently, the absence of host proviral factors results in reduced virus replication, movement, or both, or in the absence of infection. Thus, without affecting growth and development, a permissive host may be transformed into a nonhost by mutating/editing or inactivating proviral factors that determine virus susceptibility [33,129]. This concept creates a remarkable opportunity to engineer resistance to viruses through gene editing [129,130,131]. An important part of the process is the identification and characterization of susceptibility genes to plant virus infection.

Factors nonessential for host survival and with proviral activity have been identified for all stages of the virus infection cycle (Table 1). Host factors necessary for infection by DNA viruses, negative-strand, and dsRNA viruses are underrepresented. Given the agricultural importance of geminiviruses, tomato spotted wilt virus, and other orthotospoviruses, this knowledge gap is a research opportunity with important practical applications. Additionally, identification and characterization of new proviral factors is expected to improve our understanding of basic mechanisms governing virus–host interactions.

Which specific plant factors are the most promising gene editing targets? Likely, the answer will need to be determined for each plant–virus combination or by virus groups. Under this scenario, and for practical applications, an important future challenge is the identification of proviral factors required by groups of viruses of agricultural importance, such as potyviruses, orthotospoviruses, or geminiviruses. In a complementary or alternative approach, identification of host factors could be directed to particular diseases, such as maize lethal necrosis [132,133,134], cassava brown streak disease, or cassava mosaic disease [135]. Another important challenge is the assessment of the risk of viruses mutating in order to adapt to hosts with mutant proviral factors.

The identification of host factors that determine susceptibility to plant viruses in combination with gene editing provides a valuable tool to engineer genetic resistance to viruses and to understand the basic mechanisms of plant–virus interactions.

## Figures and Tables

**Table 1:** Representative nonessential host factors that condition susceptibility to plant viruses.

Host Factor	Cellular Function	Virus and Viral Factor	Host System	Technique	Reference
Viral RNA translation					
DED1-18	General translation	BMV RNA2	Yeast	Genetic screen	[48]
RISP and eIF3	Activation of polycistronic mRNA translation	CaMV TAV	Yeast	Yeast two-hybrid	[50]
eIF4G and eIF4G2	Translation initiation	LMV and PPV VPg	*A. thaliana*	Genetic analysis	[51]
LSM1-7 and PATH1	Deadenylation-dependent mRNA decapping	BMV, RNA1, RNA2, and RNA3	Yeast	Genetic analysis of single-mutant strains	[10,49]
Virus replication complex formation					
PEX19	Transport of membrane proteins to the peroxisome	TBSV p33	Yeast	Immuno-precipitation	[52]
ESCRT proteins	Membrane bending/budding away from the cytoplasm	TBSV p33	Yeast *N. benthamiana*	Genome-wide screen	[53]
		BMV 1a	Yeast	Genetic analysis of single-mutant strains	[54]
RHP	Induce positive curvature to peripheral ER membranes	BMV 1a	Yeast	Genome-wide screen	[55]
SYP71	Membrane fusion between transport vesicles and target membranes	TuMV 6K2	*A. thaliana*	Colocalization with the virus replication complex	[56]
ACBP	Lipid biosynthesis	BMV 1a	Yeast	Genome-wide screen	[57]
RAB5	Regulation of endosomal biogenesis	TBSV p33	Yeast *N. benthamiana*	Genome-wide screen	[58]
SYP81	Peroxisome protein distribution	TBSV p33	Yeast *N. benthamiana*	Yeast two-hybrid	[59]
Chl-PGK	Glycolytic, glucogenic, and photosynthetic pathways	BaMV RNA (3′ UTR)	*N. benthamiana*	Electrophoretic mobility shift and mass spectrometry	[60]
ERV14	ER vesicle formation	BMV 1a	Yeast	Yeast GFP-tagged library	[61]
ARF1	Formation of coat protein complex I vesicles on Golgi membranes	RCNMV p27	*N. benthamiana* *A. thaliana*	Affinity purification and mass spectrometry	[62]
ERO1	Disulfide bond formation within the ER lumen	BMV 1a	Yeast	Genetic analysis	[63]
eEF1A	Translation elongation and unfolded protein response	TMV 126K and 3′ UTR of genomic RNA	*N. benthamiana*	Virus-induced gene silencing	[64]
		TuMV NIb	*A. thaliana*	Tandem affinity purification	[65]
		TBSV RdRp	Yeast	Proteomics	[66]
		BaMV RNA (3′ UTR)	*N. benthamiana*	Electrophoretic mobility shift and mass spectrometry	[67]
		TMV 126K and genomic RNA	*N. benthamiana*	Virus-induced gene silencing	[64]
		TYMV 3′ UTR	*Vigna unguiculata*	Luciferase assays in protoplasts	[68]
Accumulation or activity of the replication proteins					
LSM1	Decapping and degradation of cytoplasmic mRNAs	BMV 1a	Yeast	Yeast UV mutagenesis and genetic analysis	[69]
OLE1	Conversion of saturated to unsaturated fatty acids	BMV 2a	Yeast	Yeast UV mutagenesis and genetic analysis	[70]
GAPDH	Glycolysis and gluconeogenesis	TBSV p33	Yeast *N. benthamiana*	Affinity purification and mass spectrometry	[71]
HSP70 and HSP90	Protein folding, refolding, ubiquitination, regulation of transcription	RCNMV p27	*N. benthamiana*	Affinity purification and mass spectrometry	[72,73]
HSP70		TBSV p33	Yeast	Proteomics	[74,75]
HSC70-2		BBSV p23 and CP	*N. benthamiana*	Immuno-precipitation and mass spectrometry	[76]
AtRH8 PpDDXL	mRNA processing	TuMV VPg	*A. thaliana Prunus persica*	Yeast two-hybrid	[37]
AtRH9	RNA metabolism	TuMV NIb	*A. thaliana*	Genetic analysis of single-gene mutants	[77]
PABP2 PABP4 PABP8	Translation initiation	TuMV VPg and NIb	*A. thaliana*	Copurification and genetic analysis	[78,79]
TOM1, TOM2, ARL8	Integral components of membranes	TMV-Cg, ToMV, 130K, and 180K	*N. benthamiana A. thaliana*	Sucrose gradient sedimentation and affinity purification	[80,81]
Virus movement					
eIF(iso)4E	Translation initiation	TuMV VPg	*A. thaliana*	EMS mutagenesis	[33]
		PevMoV, PVY VPg	*Capsicum spp.*	Comparative mapping	[82,83]
		TEV VPg	*A. thaliana Capsicum* spp.	Genetic analysis and genetic complementation	[46,84]
PDL1, PDL2, PDL3	Cell-to-cell trafficking	GFLV MP and CaMV MP	*A. thaliana*	Genetic analysis	[85]
KNOLLE	Membrane fusion	GFLV MP	BY-2 cells	Immuno-precipitation	[86]
PME	Cell wall-modifying enzyme	TMV, CaMV MP	*N. tabacum*	Renatured blot overlay	[87]
MYOSIN XI-2	Organelle trafficking	TMV 126 kDa	*N. benthamiana*	Pharmacological disruption and virus-induced gene silencing	[88]
Actin	Intra- and intercellular trafficking	TMV, PVX, 126K TBSV p33	*N. benthamiana*	Pharmacological disruption and virus-induced gene silencing	[88]
FIBRILLARIN	rRNA processing, formation of cajal bodies	GRV ORF3	*N. benthamiana*	Virus-induced gene silencing	[89]
PVIP1	Maintenance of the root and shoot apical meristems	TuMV VPg	*A. thaliana*	Yeast two-hybrid	[90]
SYTA	ER-plasma membrane tethering	CaLCV MP TMV and TVCV 30K TuMV P3N-PIPO	*A. thaliana*	Yeast two-hybrid	[91,92]
PCaP1	Microtubule depolymerization	TuMV P3N-PIPO	*A. thaliana*	Yeast two-hybrid	[93]
SEC24A	Intracellular protein transport	TuMV 6K2	*A. thaliana*	Yeast two-hybrid	[19]
cPGK2	Gluconeogenesis and glycolysis	PPV, undetermined	*A. thaliana*	Genome-wide association mapping	[94]
RHD3	Generation of the tubular ER network	TSWV NSm	*A. thaliana N. benthamiana*	Genetic analysis	[47]
TOR1 TOR2	Orientation of cortical microtubules	TMV 126/183 k	*A. thaliana*	Experimental virus evolution	[95]
eEF1B	Translation elongation and unfolded protein response	PVX TGBP	*N. benthamiana*	Yeast two-hybrid, immuno-precipitation	[96]
DBP1	Proteosome-mediated regulation of eIF(iso)4E	PPV and TuMV, undetermined	*A. thaliana*	Proteomics, yeast two-hybrid, immuno-precipitation	[97]
CmVPS41	Vesicle trafficking from Golgi to the vacuole	CMV 3a	*Cucumis melo*	Fine mapping	[98]
RNA silencing suppression					
RAV2	Negative regulation of transcription	TEV HC-Pro and Carmovirus p38	*A. thaliana*	Yeast two-hybrid and immuno-precipitation	[99]
rgs-CaM	Cellular signaling	TEV HC-Pro	*N. tabacum*	Yeast two-hybrid	[100]
RH8	mRNA binding and processing	PPV and TuMV VPg	*N. benthamianaA. thaliana*	Yeast two-hybrid	[37]
Nbrgs-CaM	Cellular signaling	TYLCCV DNA satellite βC1	*A. thaliana N. benthamiana*	Transcriptional profiling	[32]
OsSAMS1	Ethylene biosynthesis	RDV Pns11	*Oryza sativa*	Yeast two-hybrid	[101]
AtRAN-F2b	Late endosome to vacuole transport	CaMV MP	*A. thaliana*	Colocalization and coprecipitation	[102]
Virion assembly					
CK2 CPIP HSP70 CHIP	Protein phosphorylation Cochaperone Protein ubiquitination Ubiquitin ligase	PVA CP	*A. thaliana N. benthamiana*	Coprecipitation	[103]
Virus accumulation					
CAT1	Decomposition of hydrogen peroxide	PepMV p26	*N. benthamiana*	Yeast two-hybrid	[104]
OsSAMS1	Ethylene biosynthesis	RDV Pns11	*Oryza sativa*	Yeast two-hybrid	[101]
RIM1	Transcription factor	RDV, undetermined	*O. sativa*	Tos17 insertional mutagenesis	[105,106]
FDH1	Catalyzes oxidation of formate into CO_2_	CMV 1a	*Capsicum annum*	Yeast two-hybrid	[107]
CTR3	Calcium binding in the ER	CMV 1a	*C. annuum*	Yeast two-hybrid	[107]
PDIL5	Protein folding	BaMMV and BaYMV	*Hordeum vulgare*	Map-based cloning	[108]
MPI7	Vesicle-mediated transport	CaMV MP	*A. thaliana*	Yeast two-hybrid	[109]
IRE1A, IRE1B and bZIP60	Unfolded protein response	TuMV 6k2	*A. thaliana*	Genetic analysis	[110]
eEF1A eEF1B	Translation elongation and unfolded protein response	SMV P3	*Glycine max*	Cellular fractionation and Yeast two-hybrid	[111]
EXA1	Adaptor that binds proline-rich sequences	PLAMV, AltMV, and PVX, undetermined	*A. thaliana*	EMS mutagenesis	[112]

**Virus names:** alternanthera mosaic virus (AltMV), bamboo mosaic virus (BaMV), barley yellow mosaic virus (BaYMV), barley mild mosaic virus (BaMMV), beet black scorch virus (BBSV), brome mosaic virus (BMV), cabbage leaf curl virus (CaLCV), cauliflower mosaic virus (CaMV), cucumber mosaic virus (CaMV), grapevine fanleaf virus (GFLV), groundnut rosette virus (GRV), lettuce mosaic virus (LMV), pepino mosaic virus (PepMV), plantago asiatica mosaic virus (PIAMV), pepper veinal mottle virus (PevMoV), plum pox virus (PPV), potato virus A (PVA), potato virus X (PVX), rice dwarf virus (RDV), red clover necrotic mosaic virus (RCNMV), soybean mosaic virus (SMV), tobacco etch virus (TEV), tobacco mosaic virus (TMV), tomato bushy stunt virus (TBSV), tomato mosaic virus (ToMV), tomato yellow leaf curl China virus (TYLCCV), tomato spotted wilt virus (TSWV), turnip mosaic virus (TuMV), turnip vein clearing virus (TVCV), turnip yellow mosaic virus (TYMV).
